# Physiological and Biochemical Responses of *Cucumis melo* L. Chloroplasts to Low-Phosphate Stress

**DOI:** 10.3389/fpls.2018.01525

**Published:** 2018-10-22

**Authors:** Pengli Li, Jinyang Weng, Qing Zhang, Liyao Yu, Qi Yao, Liying Chang, Qingliang Niu

**Affiliations:** ^1^School of Agriculture and Biology, Shanghai Jiao Tong University, Shanghai, China; ^2^Planting Management Station, Ningbo, China

**Keywords:** low-Pi stress, photoinhibition, chlorophyll fluorescence, electron transport, proton transport, ATP synthase activity, antioxidants

## Abstract

Phosphorus (P) is a limiting plant soil nutrient. Long-term low inorganic phosphate (Pi) irreversibly damages plant cells and hinders plant growth. Plants have evolved several adaptive biochemical, physiological, and developmental responses to low-Pi stress. However, little is known about chloroplast responses to low-Pi stress. In this study, we used physiological and biochemical analyses to investigate melon chloroplast responses to low-Pi stress. The results indicated that low-Pi stress impeded melon seedling growth and reduced its dry matter content by inhibiting the photosynthesis. Low-Pi stress reduced the P content in shoots, which inhibited ATP synthase (ATP-ase) activity, and disturbed the proton and electron transport efficiency on chloroplast photosynthetic electron transport chain. In addition, low-Pi stress induced reactive oxygen species (ROS) production in the leaves, which caused membrane peroxidation. Therefore, redox homeostasis was not maintained, and the melon leaves presented with symptoms of photooxidative stress. To mitigate photoinhibition, the melon plants initiated non-photochemical chlorophyll fluorescence quenching (NPQ) initiated by acidification of the thylakoid lumen to dissipate excess excitation energy, significantly improved ROS-scavenging enzyme activity. Based on these experimental results, we concluded that low Pi inhibited photosystem activity and caused photooxidative stress and photoinhibition. To alleviate these negative effects, the plant activated its NPQ mechanism, alternative electron transport pathways, and antioxidant system to protect its chloroplasts.

## Introduction

Nutrient deficiency substantially reduces crop quality and yield. Phosphorus (P) is an essential plant macronutrient required for growth and development. It participates in the synthesis of nucleotides, cell structural components, and membranes. It is also involved in membrane fluidity, photosynthesis, respiration, glycolysis, redox reactions, signal transduction, lipid metabolism, carbohydrate transport, and osmotic potential maintenance (Vance et al., 2003; Abel et al., 2010; Puga et al., 2017; Wang et al., 2017). The dominant form of phosphorus used by plants is inorganic phosphate (Pi). It is abundant in the soil but is often unavailable in terrestrial ecosystems because it makes insoluble compounds with Fe^3+^, Al^3+^, Ca^2+^, and Mg^2+^ (Raghothama and Karthikeyan, 2005a,b). Therefore, >30% of the arable lands worldwide require Pi fertilizer application. These amendments incur resource, financial, and labor force costs (Fita et al., 2011). Pi deficiency is especially severe in the agricultural soil of China (Han et al., 2005). In contemporary farming, mineral Pi fertilizers are widely used and have realized substantial economic benefits. However, their excessive use causes aquatic eutrophication and hypoxia and, ultimately environmental degradation. Moreover, the Pi rock used as the source material for most Pi fertilizers is non-renewable. It is estimated that Pi rock reserves will be depleted by 2050 (Vance et al., 2003; Cordell et al., 2009). It is, therefore, important to understand plant Pi sensitivity and the adaptive mechanisms in plants responding to low-Pi stress.

To cope with environmental Pi deficiency, plants evolved various morphological, physiological, and biochemical responses. These may enhance soil Pi uptake, remobilization of Pi within the plant body by modifying root architecture, root organic acids and acid phosphate exudation, induction of high-affinity Pi transporters, and root-microbial symbioses (Niu et al., 2013; Zhang et al., 2014a; Scheible and Rojas-Triana, 2015; Vengavasi et al., 2017). Analyses of the molecular mechanisms underlying phosphate sensing, signaling, and adaptation in plants showed that Pi deficiency is locally perceived by the root system. Root-to-shoot and shoot-to-root signals coordinate Pi deficiency responses at the whole-plant level. Chromatin remodeling and transcriptional and posttranslational modifications globally regulate numerous Pi deficiency responses (Zhang et al., 2014; Li H. et al., 2016). Transcriptomic and proteomic studies identified many genes and proteins were directly affected by Pi deficiency conditions. These genes/proteins participate in photosynthesis, carbohydrate metabolism, energy metabolism, secondary metabolism, signal transduction, protein synthesis, cell rescue, cell defense, and virulence (Zhang et al., 2014c). Most experiments focused on the roots of model plants like Arabidopsis, maize, rice, and soybean (George et al., 2010; Chiou and Lin, 2011; Gruber et al., 2013). However, detailed information on the effects of low-Pi stress on specific processes/compartments/machineries is still lacking, particularly for aerial plant organs like the leaves.

Photosynthesis is a multi-step cooperative process occurring in the chloroplasts. It consists of a light (photo) reaction and a dark reaction. The photoreactions are mediated by the protein complexes PSII, Cytb_6_f, PSI, and ATP synthase located in the thylakoid membranes. These absorb, transport, and convert light energy into ATP and NADPH which, in turn, drive CO_2_ assimilation in the stroma (Rochaix, 2011). Earlier studies on soybean, maize, and other crops showed that Pi starvation inhibited photosynthesis (Fredeen et al., 1990; Usuda and Shimogawara, 1992; Chu et al., 2018). It was believed that the phosphate deficiency targeted the dark reaction by decreasing ATP and NADPH, activating Rubisco, and regenerating RUBP. Recent proteomic data on maize leaves revealed that low-Pi treatment inhibited photosynthesis by downregulating the proteins involved in CO_2_ enrichment, the Calvin cycle, and the electron transport chain (ETC) (Zhang et al., 2014c). It remains to be determined how downregulation of these proteins is reflected in the key protein complexes facilitating photoreaction. To our knowledge, no experimental data has yet been reported for the response of chloroplasts to low-Pi stress in melon. Therefore, it is uncertain how the Pi level influences the photosystems in higher plants.

Melon (*Cucumis melo* L.) is a very popular fruit worldwide. According to recent data, 1,245,841 ha were planted with melon and the total global yield was 31,166,896 tons (FAOSTAT, 2016)^1^. It is an important fruit in tropical and subtropical areas. However, these regions often have Pi-deficient soils. There is relatively little data on the effects of low-Pi stress on melon. Consequently, progress toward improving melon growth under low-Pi conditions has been impeded.

In the present study, the sensitivity of melon to low-Pi stress was investigated and the effects of Pi deficiency on photosynthesis in melon were analyzed. Low Pi retarded melon seedling development and decreased CO_2_ assimilation rates relative to the control. Low Pi also inhibited the activity of ATP-ase, photosystems II (PSII), I (PSI), non-cyclic and cyclic electron transport and promoted pseudo-ring electron transport. Phosphate deficiency stimulated photoprotective mechanisms like NPQ, alternative electron transport pathways, and ROS scavenging mechanisms like the carotenoid and ROS-scavenging enzyme systems.

## Materials and Methods

### Plant Material and Phosphate Treatment

The popular melon (*C. melo* L.) variety ‘Lvtianshi’ was selected for this study. Hydroponic experiments were conducted in the greenhouse of Shanghai Jiao Tong University, China. Seeds were germinated in a substrate composed of turf, perlite, and vermiculite (v/v/v = 9/3/1). Melon seedlings were carefully transplanted at the flat cotyledon stage to shallow-mouthed plastic trays containing 10 L of half-strength modified Hoagland’s nutrient solution consisting of 3 mM KNO_3_, 2.5 mM Ca(NO_3_)_2_, 1.0 mM MgSO_4_⋅7H_2_O, 25 μM KCl, 12.5 μM H_3_BO_3_, 1 μM MnSO_4_, 1 μM ZnSO_4_, 0.25 μM CuSO_4_, 0.25 μM H_2_MoO_4_, and 13.4 μM Fe-EDTA. The planting density was 40 seedlings/tray. The pre-experiment and certain studies indicated that a low-Pi treatment was 0.025 mM or 0.001 mM Pi whereas the control (CK) was 0.25 mM Pi (hereafter, P_0.025_, P_0.001_, and P_0.25_, respectively) (Niu et al., 2013; Cheng et al., 2014). Low-Pi media were prepared by substituting K_2_SO_4_ for KH_2_PO_4_ so that the concentration of K in the medium was 3 mM for all treatments.

Uniform 7-day-old seedlings were selected as the control (CK) or low-Pi supply treatments. There were three trays per treatment and one tray served as a biological repeat. The pH of the medium was adjusted daily to 6.4 ± 0.2 with HCl or NaOH. Solutions were changed every 3 days and trays were rearranged randomly. Plants were grown at 25°C/16°C day/night temperature with a 14-h photoperiod, an irradiance of 600 ± 20 μmol m^-2^ s^-1^, and 50–75% relative humidity. Three independent replications were carried out. After 14 days treatment, the samples were harvested for use in the physiological and biochemical assays.

### Determination of Growth Indices

Growth was evaluated by measuring plant height, stem diameter, leaf area, and dry weight. Leaf area was measured in ImageJ (Image J 1.8.0).

### Determination of Chlorophyll and Carotenoid Content

Chlorophyll (*Chl*) was extracted from 0.5 g fresh leaves in 95% (v/v) ethanol until complete bleaching. The concentration was determined by measuring extract absorbances at 470 nm, 649 nm, and 665 nm in a spectrophotometer (Hitachi U-3000; Hitachi, Ltd., Chiyoda, Tokyo, Japan). The *Chl a, Chl b*, and carotenoid (*Car*) levels were calculated according to a method described previously (Wellburn and Lichtenthaler, 1984).

### Determination of Total P Content in Tissues

The roots and shoots of the harvested samples were separated at the root-hypocotyl junction and separately dried at 105°C to constant weights. The total P content in the root and shoot tissues was determined by inductively coupled plasma atomic emission spectroscopy (ICP-AAS; ICP 7600; Thermo Fisher Scientific, Waltham, MA, United States) according to the method of Jacobsen and Fongen (1999).

### Measurement of Gas Exchange Parameters and Chlorophyll *a* Fluorescence

The gas exchange parameters were measured on the first fully expanded true leaf with a GFS-300 (HeinzWalz, Effeltrich, Germany) at 25°C, relative humidity 85%, a cuvette air flow rate of 750 mL⋅min^-1^, and an ambient CO_2_ concentration. A combination of red and blue LEDs emitted a PPFD of 600 μmol⋅m^-2^⋅s^-1^. Measurements were taken once per leaf. There were four different leaves per replicate.

IMAGING-PAM and DUAL-PAM-100 measuring systems (HeinzWalz, Effeltrich, Germany) were used to measure chlorophyll *a* fluorescence, PQ pool, and post-illumination rise as described by Zhang et al. (2014b) and Huang et al. (2015), with minor modifications.(Zhang et al., 2014b; Huang et al., 2015). The light-adapted curves for the fluorescence parameters were recorded after 2 min exposure to various PPFDs (0 μmol m^-2^ s^-1^, 12 μmol m^-2^ s^-1^, 37 μmol m^-2^ s^-1^, 147 μmol m^-2^ s^-1^, 337 μmol m^-2^ s^-1^, 462 μmol m^-2^ s^-1^, 802 μmol m^-2^ s^-1^, 1,077 μmol m^-2^ s^-1^, and 1,252 μmol m^-2^ s^-1^). All measurements were made at a CO_2_ concentration of ∼400 ± 10 μmol⋅mol^-1^.

### Measurement of Proton Gradient and the ATP Synthase Activity

Transthylakoid proton motive force (*pmf*), proton gradient (ΔpH), membrane potential (Δψ) and ATP synthase activity were measured automatically through extended emitter-detector modules as P515/535 with Dual-PAM-100 system (Schreiber, 2008; Carstensen et al., 2018). After longer dark times are given, the P515 displays complex relaxation kinetics for differentiation between Δψ and ΔpH components of the overall *pmf* (Cruz et al., 2001). According to Kramer and Sacksteder (1998) the relative amplitudes of Δψ and ΔpH can be estimated from the characteristic levels observed during the light-off response (Kramer and Sacksteder, 1998). The difference between the steady state signal and the “dark baseline” reflects Δψ during steady state illumination. The “undershoot” below the “dark baseline” is considered to be a measurement of the steady state of ΔpH. When light is off the accumulated protons are rapidly released from the lumen to the stroma via the ATP-ase, and there is a sudden excess of negative charge at the internal side of the membrane, which results in an inversed P515. Before measurement, samples were kept for more than 2 h in darkness. The 550–515 nm signal curves were recorded at dark–light–dark cycle by the Dual-PAM-100 software. The original difference of signals were measured in Volt units, which were transformed into ΔI/I units with the help of the calibration routine (Schreiber, 2008).

Total *pmf* was estimated from the total amplitude of the rapid decay of the electrochromic pigment shift (ECS) signal during 300 ms dark interval. The time constant of the first-order ECS relaxation (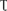
_ECS_) is inversely proportional to the proton conductivity (gH^+^) of the thylakoid membrane through the ATP-ase (Sacksteder and Kramer, 2000; Cruz et al., 2005). As a result, gH^+^ was estimated as the inverse of the decay time constant [1/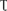
_ECS_].

The ATP-ase activity is also characterized by Rapid P515 relaxation kinetics induced by single-turnover saturating flash. P515/535 module allows to assess the essential parameters of the flash induced P515 change with single recordings. Preillumination activates the reversible ATP-ase in the thylakoid membrane, thus increasing the H^+^ conductivity of the membrane. The decay of the P515 signal reflects the relaxation of the flash induced electric field (created by charge separation in the two photosystems and electrogenic electron transport the Q-cycle at the cyt b/f complex) by H^+^ efflux via the H^+^ channel of the ATP-ase. A functionally intact photosynthetic apparatus is characterized by a fast decay after illumination (high ATP-ase activity) (Schreiber, 2008).

### MDA Content and Antioxidant Enzyme Activity Assay

Malondialdehyde (MDA) content was determined according to Siripornadulsil et al. (2002) with modifications (Siripornadulsil et al., 2002). Briefly, leaf samples (0.3 g) were homogenized with 5 mL of 5% (w/v) trichloroacetic acid (TCA) followed by centrifugation at 3,000 × *g* and 4°C for 15 min. For the assay, 0.2 mL of the supernatant was mixed with 0.5 mL of 0.5% thiobarbituric acid (TBA) solution and boiled for 10 min. The reaction was stopped by placing the mixture on ice. The mixture was then centrifuged at 3,000 × *g* and 4°C for 15 min. The absorbances of the supernatant were measured at 450 nm, 532 nm, and 600 nm relative to a blank in which the TBA was omitted to obtain the correct MDA concentration (Hodges et al., 1999).

To measure the antioxidant enzyme activity levels, crude leaf extracts were prepared by grinding the leaf samples (0.3 g) as described above and extracting them in 3 mL ice-cold 50 mM potassium phosphate buffer (pH 7.8) containing 0.2 mM EDTA and 1% (w/v) polyvinylpyrrolidone (PVP). The homogenate was centrifuged at 10,000 × *g* and 4°C for 20 min. The supernatant was used in the enzyme activity assays. Peroxidase (POD) activity was determined with methoxyphenol as the substrate and evaluated by monitoring the increase in absorbance at 470 nm (Trevisan et al., 1997). One enzyme unit corresponded to a 0.1 unit min^-1^ increase or decrease in absorbance at 470 nm. Superoxide dismutase (SOD) activity was determined by measuring the inhibition of the photochemical reduction of nitro blue tetrazolium (NBT) according to the method of Stewart and Bewley (1980). For SOD, one enzyme unit was defined as the amount of enzyme extract causing a 50% inhibition of the reaction (Beauchamp and Fridovich, 1971).

### Statistical Analysis

The experiment was a completely randomized design with three replicates. Each replicate consisted of 40 plants. One-way ANOVA was used to test for significance. Significant differences (*P* < 0.05) between treatments were determined using Duncan’s multiple range test.

## Results

### Melon Morphophysiological Traits Under Varying Pi Availability

**FIGURE 1:**
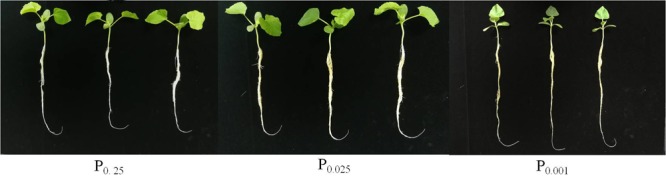
Effects of varying Pi supply on melon seedling morphology.

To explore the effect of Pi on melon seedling morphology, growth and development parameters were measured (Figure 1 and Table 1). Plant height and stem diameter significantly decreased with decreasing Pi supply. Plant height decreased by 8.81% (P_0.025_) and 30.81% (P_0.001_) and stem diameter decreased by 7.16% (P_0.025_) and 17.01% (P_0.001_) compared to those of the control (P_0.25_), respectively. Total leaf area is an indicator of the amount of photosynthetic activity available for biomass production (Muldoon et al., 1984). It was reduced by 58.09% (*P* < 0.05) relative to the control in P_0.001_. Root and shoot biomass accumulation were also substantially lower in P_0.001_ than in the control. The root/shoot ratio was significantly higher in P_0.001_ than in the control. However, the leaf area, dry weight and root/shoot ratio were not different between P_0.025_ and the control.

**Table 1:** The growth state of melon seedlings on varying Pi supply.

Pi supply (mmol.L^-1^)	Plant height (cm)	Stem diameter (mm)	Leaf area (cm^2^)	Dry weight/g		Root/Shoot
				Root	Shoot	
0.25	11.61 ± 0.77^a^	4.19 ± 0.24^a^	108.26 ± 4.13^a^	0.087 ± 0.0039^a^	0.302 ± 0.0057^a^	0.29 ± 0.016^b^
0.025	10.59 ± 0.1^b^	3.89 ± 0.22^b^	108.04 ± 4.2^a^	0.090 ± 0.0065^a^	0.293 ± 0.0029^a^	0.30 ± 0.018^b^
0.001	8.03 ± 0.57^c^	3.48 ± 0.15^c^	45.38 ± 2.89^b^	0.055 ± 0.0012^b^	0.224 ± 0.0051^b^	0.33 ± 0.020^a^

*The values represent the mean ± standard deviation (SD) and the different letters indicate significant differences among values for each Pi supply (*P* < 0.05).*

To determine whether low Pi affected leaf chlorophyll levels, the chlorophyll content in the first true leaf was measured (Table 2). The chlorophyll content in P_0.001_ was significantly higher than that for the control. Low-Pi stress also increased the carotenoid content by 22.6% (P_0.025_) and 46.7% (P_0.001_) compared with that of the control. The *Chl a*/*Chl b* and *Car/Chl* ratios were significantly higher in the low-Pi treatments than the control.

**Table 2:** Influence of varying Pi supply on leaf chlorophyll content, *Chl a*/*Chl b* and *Car*/*Chl.*

Pi supply (mM)	Chlorophyll concentration (mg g^-1^ Fw)	Carotenoid concentration (mg g^-1^ Fw)	*Chl a*/*Chl b*	*Car*/*Chl*
0.25	1.38 ± 0.06^b^	0.11 ± 0.01^c^	1.99 ± 0.16^b^	0.09 ± 0.01^b^
0.025	1.39 ± 0.12^b^	0.14 ± 0.01^b^	2.59 ± 0.16^a^	0.10 ± 0.01^a^
0.001	1.68 ± 0.04^a^	0.17 ± 0.01^a^	2.44 ± 0.10^a^	0.10 ± 0.01^a^

*The values represent the mean ± SD and the different letters indicate significant differences among values for each Pi supply (*P* < 0.05).*

**FIGURE 2:**
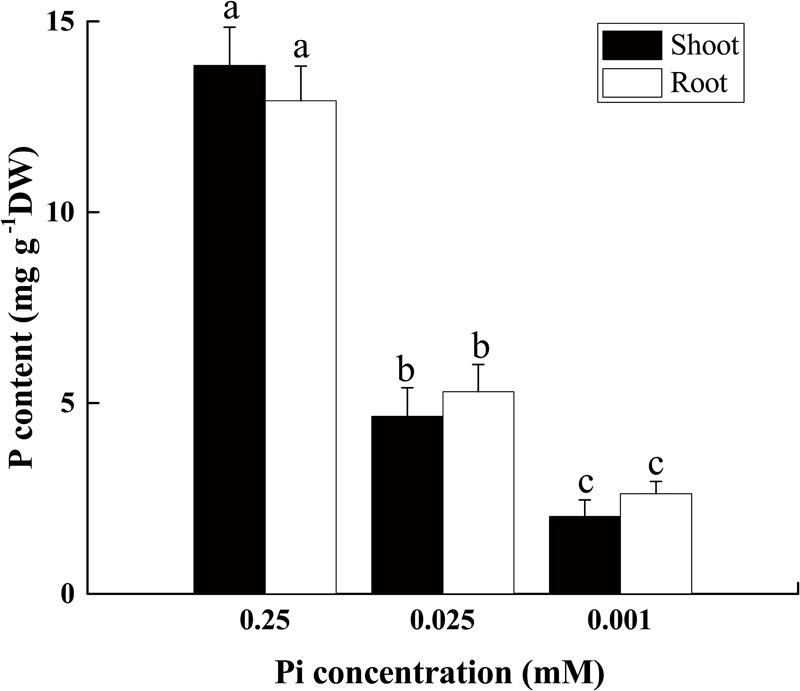
P content in shoot and root tissues of seedlings grown under varying Pi supply. All data were determined on the 14th day after low-Pi treatment. The bars (mean ± SD, *n* = 3) labeled with different letters are significantly different at *P* < 0.05 according to Duncan’s test.

The phosphorus content declined in both the root and shoot as the external Pi supply decreased from 0.25 mM to 0.025 mM or 0.001 mM (Figure 2). After 14 days of low-Pi stress, the P content in the shoot decreased by 66% (P_0.025_) and 85% (P_0.001_) relative to that of the control, respectively. In the control, the P content in the roots (12.9%) was slightly lower than that in the shoot (13.8%) (S/R ≈ 1). P_0.025_ dramatically reduced the shoot and root P content (S/R < 1). The shoot P content decreased more than that of the root. The P_0.001_ treatment even further lowered the P content in the shoots relative to that of the roots. For this treatment, the shoot P was significantly lower (22.8%) than that of the root (S/R < 1). These results indicated that low Pi redistributed P in the plant body and allocated relatively more of it to the roots. Therefore, the low-Pi treatment used in these experiments effectively induced a low-Pi stress response in *Cucumis melo* L.

### Causes of Photosynthesis Reduction in Melon Under Low-Pi Stress

**FIGURE 3:**
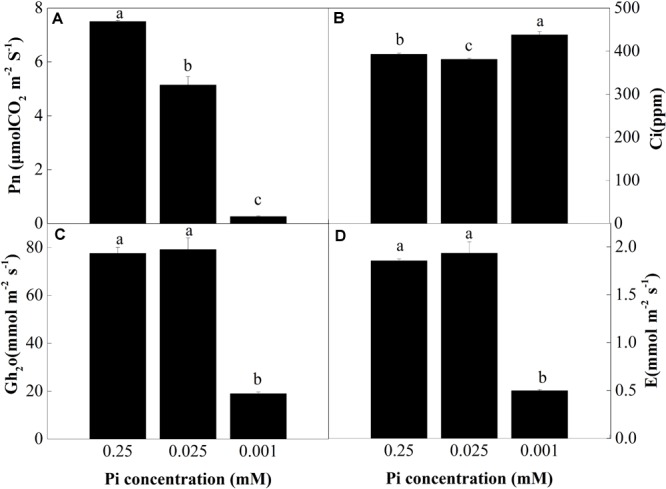
Measurement of gas exchange parameters: **(A)** net photosynthesis rate (Pn), **(B)** intercellular CO_2_ concentrations (Ci), **(C)** stomatal conductance (Gh_2_o), and **(D)** transpiration rate (E) of the first leaves. Error bars (mean ± SD, *n* = 3) labeled with lowercase are significantly different at *P* < 0.05 in Duncan’s test.

To investigate the effect of low-Pi on photosynthesis, plant gas exchange parameters were measured in the first fully expanded true leaf. Figure 3A shows that the net photosynthesis rate (Pn) decreased with decreasing Pi supply. Relative to the control, Pn for the P_0.025_ and P_0.001_ treatments were 31.4% and 96.4% lower than that for the control, respectively. There were significant differences in the intercellular foliar CO_2_ concentrations (Ci) between the control and the low-Pi plants (Figure 3B). The intercellular CO_2_ concentration (Ci) in the P_0.001_ treatment was significantly higher than those for the control and the P_0.025_ treatment. The stomatal conductance (G_h2o_) and transpiration rate (E) in the P_0.001_ treatment were significantly lower than those for the control and P_0.025_ treatments (Figures 3C,D). The Pn was significantly lower in the P_0.025_ treatment than that of the control but there were no significant differences in Ci, G_h2o_, or E between these two treatments. Therefore, P_0.025_ treatments did not inhibit stomatal opening or transpiration rate compared with the control. Stomatal limitation (Ls) was calculated on the basis of these data. The Ls were 10.76% (P_0.25_), 13.37% (P_0.025_), and 0.43% (P_0.001_). According to the very low Pn, G_h2o_, and Ls under P_0.001_, non-stomatal limitation factors were the main causes of photosynthetic rate reduction in melon under low-Pi stress.

### Effect of Low-Pi Stress on the Function of PSII in Melon Seedling

There are many non-stomatal photosynthesis limitation factors. However, we focused on the photosystem in this study. To clarify the impact of low Pi on the photosynthetic light reactions in melon, we compared chlorophyll *a* fluorescence in the first true leaves of the control and low-Pi treated plants by imaging PAM chlorophyll fluorometry (Zealquest Scientific Technology Co., Ltd., Shanghai, China).

**FIGURE 4:**
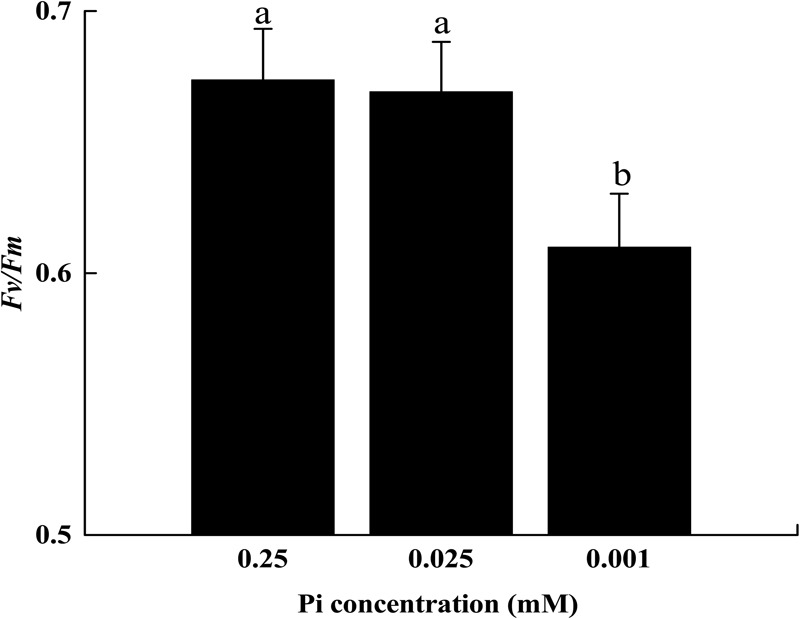
The maximum quantum yield (*F*_v_*/F*_m_) of leaf grown under Pi conditions. Experiments were repeated three times with similar results. Error bars (mean ± SD, *n* = 3) labeled with lowercase are significantly different at *p* < 0.05 in Duncan’s test.

The initial *F*_v_*/F*_m_ measurements suggested that 14 days of low-Pi stress disturbed PSII activity in melon seedlings. The P_0.001_ treatment decreased the maximal quantum efficiency (*F*_v_*/F*_m_) of PSII by 8.9% relative to that of the control (Figure 4).

**FIGURE 5:**
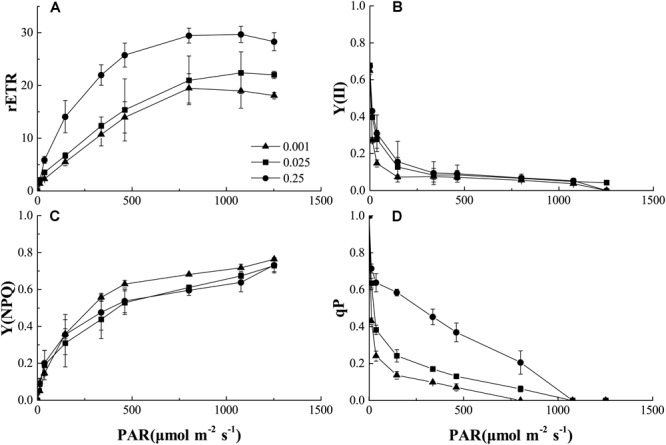
Comparisons of chlorophyll fluorescence parameters: **(A)** Plots of rETR (relative photosynthetic electron transfer rates) vs. photosynthetic active radiation (PAR). **(B)** Plots of Y (II) (PSII actual quantum yield) vs. PAR. **(C)** Plots of Y(NPQ) (non-photochemical quenching) vs. PAR. **(D)** Plots of qP (photochemical quenching) vs. PAR. Error bars represent SD of the mean (*n* = 3).

We also analyzed the light-response curves of the relative electron transport rate (rETR), actual quantum yield [Y(II)], non-photochemical quenching (NPQ), and photochemical quenching (qP) of PSII in melon under normal or low-Pi supply. Low Pi clearly lowered rETR compared to that of the control (Figure 5A). Therefore, PSII function was impaired under low-Pi stress. Y(II) reduction was more pronounced under low light intensity (PAR < 500 μmol m^-2^ s^-1^) than it was under normal light levels. Y(II) also decreased with decreasing external Pi supply (Figure 5B). However, light intensities>500 μmol m^-2^ s^-1^ repressed light quantum production. Low-Pi stress reduced Y(II) as well. NPQ dramatically increased under P_0.001_ relative to the control (Figure 5C). Therefore, excess excitation energy was dissipated as heat energy under low-Pi stress. This response may be a mechanism by which the seedlings are protected from damage under low-Pi supply. Photochemical quenching (qP) substantially decreased in the low-Pi treatments when compared with the control (Figure 5D). Therefore, low Pi inhibited photosynthetic activity and, to some extent, PSII impairment repressed photosynthesis.

### Low-Pi Stress Significantly Increased PQ Pools in Melon Seedlings

**FIGURE 6:**
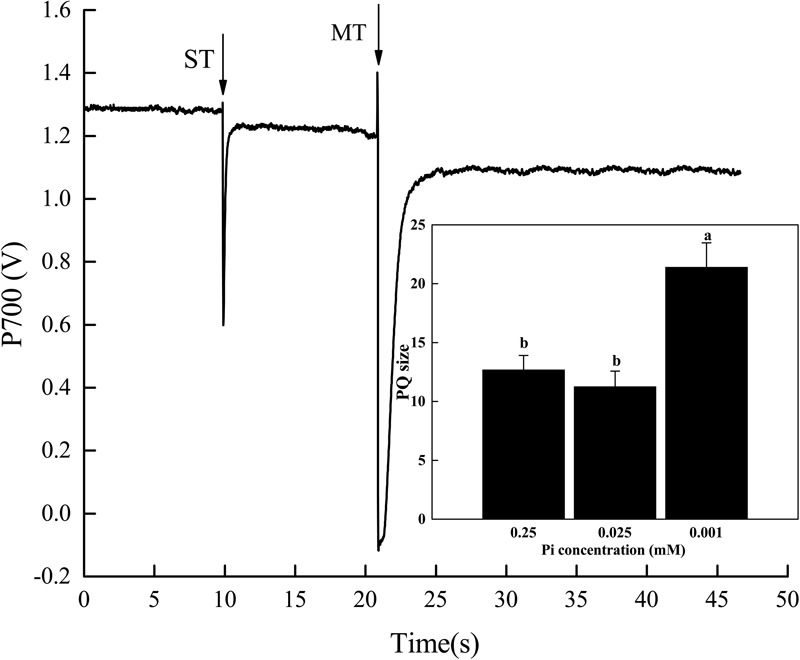
Effect of low-Pi on PQ pools of melon seedlings. The P700 signal was determined during a single turnover flashes (ST, 50 ms, PQ pool being oxidized) followed by multiple turnover flashes (MT, 50 ms, PQ pool is fully reduced) in the presence of far-red (FR) background light. The ratio of MT-area/ST-area is used to estimate the size of functional PQ pools. Error bars (mean ± SD, *n* = 3) labeled with lowercase are significantly different at *P* < 0.05 in Duncan’s test.

Relative to the control, the PQ pool size significantly increased in melon seedlings under P_0.001_ (Figure 6). The PQ pool size under P_0.001_ was 1.7× and 1.9× greater than those of the control and P_0.025_ treatments, respectively. Therefore, the electrons generated by photolysis and transported to PSII and PQ were reduced. In this way, the functional PQ pool increased. From another perspective, the electron transport efficiency of PSII diminished. This increase in the functional PQ pool may be an adaptive response to low-Pi stress.

### Effect of Low-Pi Stress on the Activity of PSI in Melon Seedling

**FIGURE 7:**
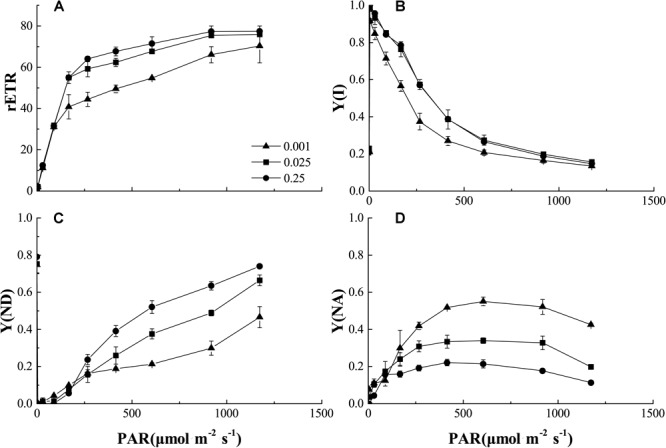
Comparisons of PSI excitation energy distribution from control and low-Pi treated seedlings. **(A)** Plots of rETR (relative photosynthetic electron transfer rates) vs. photosynthetic active radiation (PAR). **(B)** Plots of Y(I) (PSI actual quantum yield) vs. PAR. **(C)** Plots of Y(ND) (non-photochemical quenching caused by PSI donor-side limitation) vs. PAR. **(D)** Plots of Y(NA) (non-photochemical quenching caused by PSI acceptor-side limitation) vs. PAR. Error bars represent SD of the mean (*n* = 3).

In the process of photoreaction, the electrons produced by photolysis were transported from PSII to PSI. Figure 7 showed the light intensity dependence of the relative electron transport rate through PSI [rETR (I)], actual quantum yield [Y(I)], non-photochemical quenching (ND) caused by PSI donor-side limitation, and non-photochemical quenching (NA) caused by PSI acceptor-side limitation in the control and low-Pi stress treatments. The rETR (I) increased with increasing light intensity within a certain range (PAR < 500 μmol m^-2^ s^-1^), then reached steady-state (Figure 7A). The rETR(I) and Y(I) were significantly decreased by P_0.001_ stress. Y(I) decreased with increasing PAR. Therefore, increasing light intensity was not conducive to light quantum generation. Y(I) was significantly decreased by P_0.001_ stress (Figure 7B). These results suggest PSI activity was decreased by low-Pi stress. Relatively higher Y(ND) indicates that the plants received stronger light intensities but could still protect themselves by dissipating the excess light energy. Y(ND) and Y(NA) showed that under low-Pi conditions, the photoprotective capacity of PSI was significantly reduced and the plants were more vulnerable to injury caused by excess illumination. We speculate that the decreases in light-harvesting efficiency and the photoprotective capacity of PSI partially accounted for the decreased photosynthetic rates under low-Pi stress.

### Effect of Low-Pi Stress on Cyclic Electron Transport Around PSI in Melon Seedling

**FIGURE 8:**
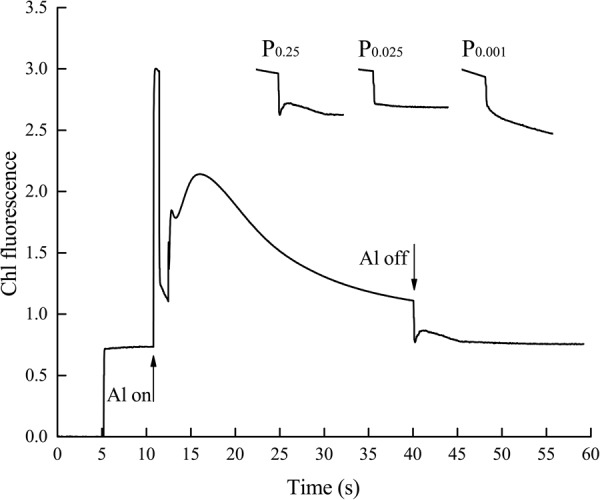
Effects of low-Pi on post-illumination increase of Chl fluorescence in the first true leaf. The curve showed a typical trace of Chl fluorescence in the first true leaf. The leaf was exposed to actinic light (AL) (587 μmol m^-2^ s^-1^) for 30 s. AL was turned off and the subsequent change in Chl fluorescence level was monitored. After switching the AL off, transient increases in Chl fluorescence were recorded under low measure light. Insets were magnified traces from the time of 35–50 s area.

The aforementioned results indicate that non-cyclic electron transport was significantly inhibited by low-Pi stress. Low-Pi stress also reduced cyclic electron transport efficiency. The chloroplast NAD(P)H dehydrogenase (NDH) complex participates in cyclic electron flow around PSI (Shikanai et al., 1998; Munekage et al., 2004). NDH activity is monitored as a post-illumination rise in chlorophyll fluorescence (Figure 8) because of the dark reduction of the plastoquinone (PQ) pool by the NDH complex (Shikanai et al., 1998). The transient increase in the basal level of chlorophyll fluorescence in the control seedlings after light-to-dark transition is explained by PQ and QA reduction via NDH complex activity. Seedlings under P_0.025_ and P_0.001_ showed no increase in fluorescence after actinic light (AL) illumination. Therefore, the NDH complex was impaired, and cyclic electron transport around PSI was inhibited by low-Pi stress.

### The Effect of Low-Pi on the Photosynthetic Proton Transport

**FIGURE 9:**
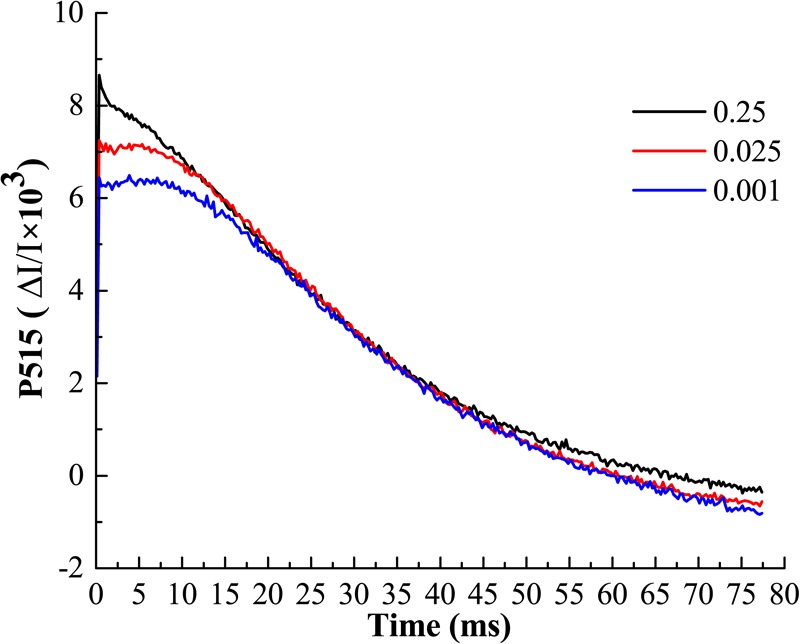
Typical recordings of P515 changes induced by saturating single turnover flashes in melon leaf. Display with 100 points averaging. A fast decay of P515 signal after illumination represents the high ATP-ase activity.

The buildup of transthylakoid ΔpH is associated with the proton transfer that is tightly coupled with photosynthetic electron transport (Kramer et al., 2003). The measurements of electrochromic bandshift (ECS, P515) represent the non-invasive way to estimate the processes related to H^+^ transport through the thylakoid membranes (Kanazawa and Kramer, 2002). In order to clarify whether the low-Pi has negative effect to photosynthetic proton transport, the *pmf*, Δψ, ΔpH and gH^+^ were determined *in vivo* (Table 3). We found that *pmf*, Δψ and ΔpH were significantly increased by P_0.001_ relative to that for P_0.25_ and P_0.025_, which indicated that low-Pi caused the acidification of thylakoid lumen. The analysis of fast ECS decay showed the significant differences in values of thylakoid proton conductance (gH^+^) (Table 3 and Figure 9). Low-Pi inhibited the ATP-ase activity, retarded proton efflux from the thylakoid lumen and increased *pmf*.

**Table 3:** Influence of varying Pi supply on photosynthetic proton transport.

Pi supply (mM)	pmf (V)	Δψ (V)	ΔpH (V)	gH^+^ (s^-1^)
0.25	0.0274 ± 0.0057^b^	0.0353 ± 0.0046^b^	0.0829 ± 0.0069^b^	10.54 ± 0.52^a^
0.025	0.0289 ± 0.0031^b^	0.0496 ± 0.0178^b^	0.1001 ± 0.0093^b^	8.67 ± 0.44^b^
0.001	0.0349 ± 0.0048^a^	0.0637 ± 0.0089^a^	0.1281 ± 0.0309^a^	8.13 ± 0.15^c^

*The values represent the mean ± SD and the different letters indicate significant differences among values for each Pi supply (*P* < 0.05).*

### Increased ROS-Scavenging Enzyme Activity Protected Chloroplasts Under Low-Pi Stress

**FIGURE 10:**
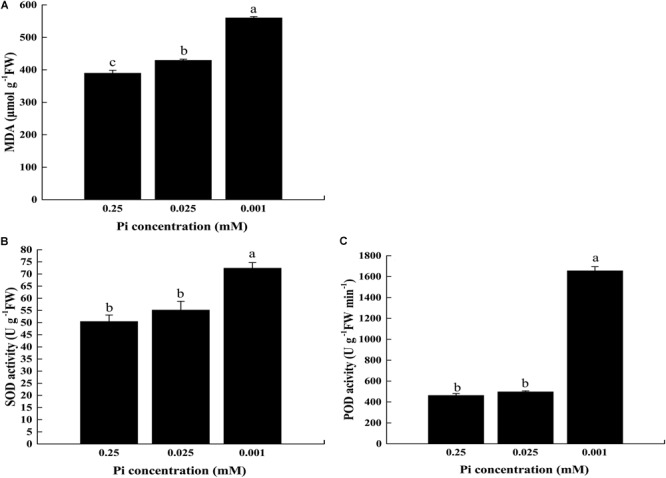
Measurement of MDA content and activity of ROS scavenging enzymes in the leaves under varying Pi supply. **(A)** Malondialdehyde (MDA) content, **(B)** superoxide dismutase (SOD) activity, and **(C)** peroxidase (POD) activity. Error bars represent SD of the mean (*n* = 3).

We estimated the extent of lipid peroxidation by measuring MDA in the first true leaf of the control and low-Pi stress plants (Figure 10A). The MDA content under P_0.025_ and P_0.001_ were 1.10× and 1.44× higher than the control, respectively, and these differences were significant. Therefore, lipid peroxidation of the thylakoid membranes was pronounced under low-Pi stress.

We measured ROS-scavenging enzyme [superoxide dismutase (SOD) and peroxidase (POD)] activity in melon under low-Pi stress. Figure 10 showed that SOD activity was increased with decreasing Pi supply (Figure 10B). For POD, the highest activity (1,656.13 U⋅g^-1^ FW⋅min^-1^) was detected in P_0.001_ treatment. Under P_0.025_, POD activity was slightly higher than that of the control (498.64 U⋅g^-1^ FW⋅min^-1^ and 463.17 U⋅g^-1^ FW⋅min^-1^, respectively) but the difference was not significant (Figure 10C). These results showed that the ROS-scavenging enzyme activity was enhanced in the leaves of melon plants subjected to low-Pi stress in order to protect the chloroplasts against photo-oxidative damage.

## Discussion

Plants require micronutrients and macronutrients during growth and development in order to have a normal and healthy physiological state and to maintain photosynthesis (Smethurst et al., 2005; Osman, 2013; Li H. et al., 2016). Previous studies have shown that Pi deficiency inhibits photosynthesis (Christoph and Simon, 1987; Furbank et al., 1987; Jacob and Lawlor, 1992; Singh and Raja, 2011; Zhang et al., 2014,c). Nevertheless, our knowledge of the response of melon chloroplasts to low-Pi stress remains limited. In this study, we examined the responses of photosynthesis and the photosystem in melon to low-Pi stress.

### Low-Pi Inhibited Photosynthesis by Non-stomatal Limitation

Photosynthesis is highly sensitive to low-Pi stress because it requires Pi to express the genes and proteins involved in the process (Zhang et al., 2014,c; Li L. et al., 2016). In the current study, low-Pi stress inhibited photosynthesis and biomass accumulation in melon seedlings. These findings corroborate those of previous studies (Xing and Wu, 2014; Zhang et al., 2014,c).

Since Pn and Ls were decreased but Ci increased under low-Pi stress in the present study (Figures 3A,B), our results indicate that non-stomatal limitation factors significantly affect photosynthesis during phosphorus deficiency (Farquhar and Sharkey, 1982; Reich et al., 2009; Suriyagoda et al., 2010). Under low-Pi conditions, *F*_v_*/F*_m_ was substantially reduced in melon under low-Pi stress. Therefore, the PSII reaction centers were affected by the Pi deficiency. Depressed rETR and Y in PSII and PSI decrease the efficiencies of both cyclic electron transport (CET) and non-cyclic electron transport (Figures 5, 7, 8). In addition, low-Pi inhibited the ATP-ase activity and proton transport (Figure 9 and Table 3). Consequently, ATP generation decreased relative to the control (Lauer et al., 1989; Fredeen et al., 1990; Carstensen et al., 2018). Low-Pi stress may have diminished the protein complex in the ETC (PSII, Cytb_6_f, PSI) (Zhang et al., 2014c). Under low-Pi stress, the constituents of the dark reaction may have repressed ATP and NADPH biosynthesis (Heber et al., 1989; Wu et al., 2006; Carstensen et al., 2018). Therefore, Pi deprivation restricted photosynthesis by reducing both ETC efficiency and ATP generation.

### Mechanisms of Alleviating Photo-Oxidative Stress Induced by Low-Pi in Melon

Low-Pi stress diminishes the capacity of the plant to harvest light and causes photoinhibition. Consequently, low-Pi stress decreases the electron transport rate, the maximum and actual PS quantum yields and ATP-ase activity (Figures 5, 7, 9 and Table 3). These are symptoms of photo-oxidative stress (Weng et al., 2008; Zhang et al., 2014c). To counteract it, plants evolved several protective mechanisms including xanthophyll-dependent dissipation of excess excitation energy (NPQ mechanism) (Figures 5C, 7C), ROS quenching/scavenging (Figures 10B,C), alternative electron transport pathways and improving the chloroplast pigment content (Table 2).

### NPQ Mechanism Was the First Line of Defense Against Pi Stress-Induced Photo-Oxidative Injury

Some of the photon flux absorbed by the antenna pigments is converted to redox energy via the ETC and used to fix CO_2_. The balance is dissipated as heat and fluorescence (Gilmore, 1999). The ΔpH component of *pmf* is the key regulatory signal for initiation of NPQ of excitation energy, which is important for photoprotection (Kanazawa and Kramer, 2002). NPQ dissipate the excess energy absorbed by the light-harvesting complex II (LHCII) in the form of heat (Lambrev et al., 2012). In the present study, the high Y(NPQ) measured under low-Pi stress (Figure 5C) suggests that the photochemical energy conversion and protective regulatory mechanisms still quenched the light energy harvested by the plants even though the leaves absorbed excess light energy. NPQ modulation in response to changing Pi levels occurs predominantly by alterations in the conductivity of the ATP-ase to protons (*g*H^+^). Decreasing *g*H^+^ will increase transthylakoid proton motive force (*pmf*), thus lowering lumen pH and contributing to the activation of NPQ (Kanazawa and Kramer, 2002). Earlier studies have indicated that NPQ is associated with the regulation of the photoprotective mechanism against high-light stress (Lambrev et al., 2012; Cui et al., 2017).

### ROS-Scavenging Enzymes Are the Second Line of Defense Against Pi Stress-Induced Photooxidative Injury

The activities of the ROS-scavenging enzymes SOD and POD were significantly increased by P_0.001_ stress (Figures 10B,C). ROS are metabolic by-products and play important roles in cell signaling and redox homeostasis maintenance. At optimal concentrations, ROS may actually be protective but at superoptimal levels they can damage lipids, proteins, and nucleic acids and cause irreversible injury (Hernández and Munné-Bosch, 2015). The results of the current study indicated a substantial degree of lipid peroxidation (based on MDA levels) in the first true leaf of melon plants under low-Pi stress (Figure 10A).

To control ROS levels and alleviate their harmful effects, plants evolved a system of defenses. Antioxidant enzymes like SODs and ascorbate peroxidases (APXs) catalyze redox reactions. Non-enzymatic water-soluble antioxidant compounds like glutathione (GSH) and ascorbate provide electrons. Non-enzymatic lipid-soluble antioxidant compounds like vitamin E and carotenoids regulate ROS levels in photosynthetic membranes and protect them from lipid peroxidation (Miret and Munnébosch, 2015; Li H. et al., 2017). These antioxidant mechanisms interact with each other in parallel or sequentially to limit the generation of peroxidized by-products more effectively (Hernández and Munné-Bosch, 2015). Several studies reported and increases in water-soluble antioxidant levels and antioxidant enzyme activities in plants under Pi deficiency. These changes also occur at the gene expression level (Weng et al., 2008; Lei et al., 2011; Zhang et al., 2014,c). Therefore, under low-Pi stress, ROS-scavenging enzyme activity increases to protect chloroplasts from low-Pi-induced photo-oxidative damage and to maintain redox homeostasis.

### Alternative Electron Transport Pathways Are Another Adaptive Response to Low-Pi Stress

**FIGURE 11:**
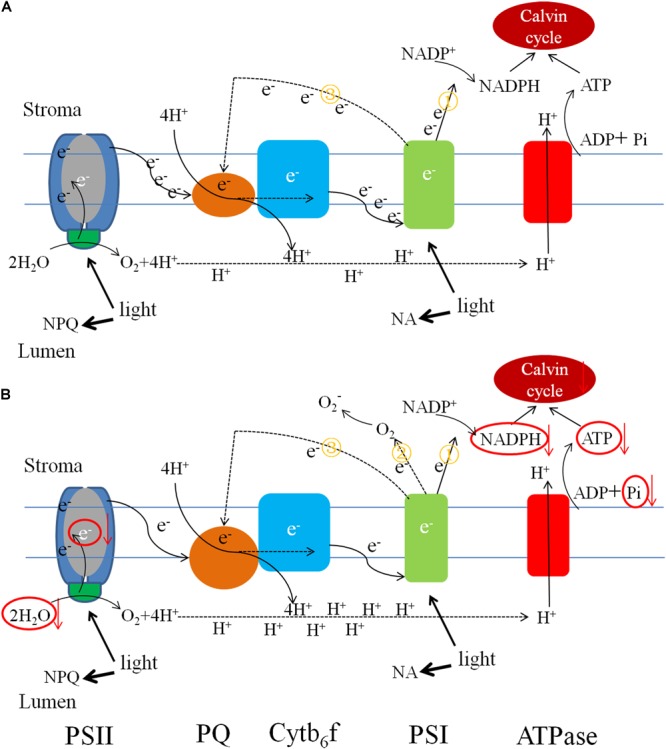
Pictorial representations of photosynthetic electron transport changes under the different Pi supply. **(A)** Electron low under sufficient Pi supply and **(B)** electron low under low Pi supply. Low Pi inhibited non-cyclic (①) and cyclic electron transport (③) around PSI, carbon fixation capacity, promoted pseudo-cyclic (②) electron transport and accumulation of H^+^ causing lumen acidification. The size of units and font represents the activities of the organelle components.

Alternative electron transport pathways alleviate the excitation energy pressure accumulated in PSII reaction centers (Zhang et al., 2014,b). In low-Pi-induced photoinhibition, excess electrons are transported to molecular oxygen and generate ROS like O_2_^-., 1^O_2_, H_2_O_2_, OH, and others at their respective reaction sites. The production of these ROS can trigger an increase in the activity levels of ROS-scavenging enzymes. Excess ROS blocks the ETC by inducing protein degradation and impeding PSII repair (Allakhverdiev et al., 2008). The results of the current study indicated that low-Pi stress inhibits both non-cyclic and cyclic electron transport but promotes pseudo-cyclic electron transport which leads to the production of O_2_^-.^ in PSI (Figure 11).

However, low-Pi stress improved the chlorophyll and carotenoid contents in leaves, which was different from the responses of barley and soybean to low-Pi stress (Frydenvang et al., 2015; Chu et al., 2018). We found that the leaf water content and free water content in low-Pi were significantly low relative to the control (the data wasn’t represented). It was assumed that low-Pi decreased the water content of the leaf to improve the P content and chloroplast pigments content.

## Conclusion

We ascertained that in melon seedlings, 0.025 mM Pi and 0.001 mM Pi constitute moderate and severe low-Pi stress, respectively. However, most of the growth indicators and photosynthesis parameters did not significantly differ between the moderate low-Pi stress treatment and the control. Therefore, 0.025 mM Pi was the optimal phosphate content for melon seedling growth. At this low level of Pi deficiency stress, seedling growth was not inhibited and certain adaptive responses were activated. Severe low-Pi stress decreased the ATP-ase activity, electron and proton transport activity, inhibited chloroplast membrane development, and induced lipid peroxidation. All of these created an imbalance between light interception and the ability of the plant to process it. The resultant effect was photoinhibition. In this state, the NPQ mechanism, ROS-scavenging enzymes, and alternative electron transport pathways were activated to reduce photooxidation induced by low-Pi stress. When these protective mechanisms fail to maintain redox homeostasis in the leaves, the plants may present with photooxidative stress symptoms like lipid peroxidation, delayed leaf expansion, and growth retardation.

## Author Contributions

PL and QN designed the research. PL, JW, QZ, LY, and QY performed the research. PL and LY analyzed the data. PL, LC, and QN wrote and revised the paper.

## Conflict of Interest Statement

The authors declare that the research was conducted in the absence of any commercial or financial relationships that could be construed as a potential conflict of interest.

## Footnotes

1 http://www.fao.org/faostat/zh/#data/QC
